# Separate Electrograms and Morphologically Distinct QRS Complexes During Ventricular Tachycardia: Critical Versus Noncritical Isthmus

**DOI:** 10.19102/icrm.2026.17081

**Published:** 2026-08-15

**Authors:** Ozcan Ozeke, Dursun Aras, Serkan Topaloglu

**Affiliations:** 1 Department of Cardiology, University of Health Sciences, Ankara Bilkent City Hospital, Ankara, Turkey; 2 Department of Cardiology, İstanbul Medipol University, Istanbul, Turkey

**Keywords:** Dual exit, multiple exit, ventricular tachycardia

## Abstract

The electrogram morphology and its timing within the ventricular tachycardia (VT) cycle enable electrophysiologists to infer the location of the scar and its relationship to the VT circuit. This becomes particularly important in the presence of double-entrance or double-exit configurations, where distinguishing the critical isthmus from a noncritical bystander is essential.

## Case presentation

**Figure 1:**
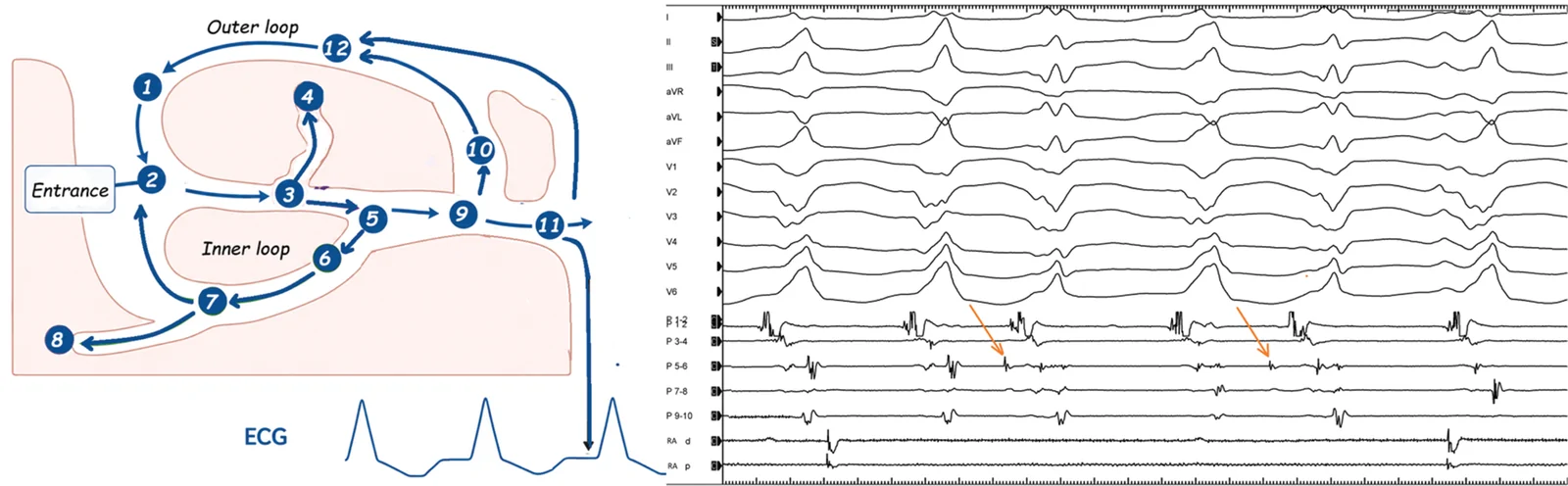
Intracardiac electrograms during ventricular tachycardia. *Abbreviation:* ECG, electrocardiogram.

The patient was a 77-year-old woman known for ischemic cardiomyopathy with a left ventricular ejection fraction of 25%, a history of sustained monomorphic VT, and an implantable cardioverter-defibrillator. EGMs recorded along the diastolic pathway using the PentaRay™ catheter (J&J MedTech, New Brunswick, NJ, USA) intermittently demonstrated mid-diastolic potentials (MDPs) that preceded each morphologically distinct QRS complex (red arrows in **Figure 1**). To better understand the mechanism of the tachycardia, a detailed analysis of the intracardiac electrograms was performed.

## Discussion

Multiple VT morphologies were observed in a majority (40%–80%) of post-myocardial infarction patients who underwent catheter ablation.^1,2^ Miller et al. described possible mechanisms that could explain variable QRS morphologies on a surface electrocardiogram, including different expressions of the same VT circuit due to preferential exit points along a shared isthmus, distinct circuits closely placed, and widely unconnected arrhythmogenic sites.^2^ Bimorphic VT is one of the most commonly known prototypes of electrical alternans and is characterized by a rapid, wide-complex electrocardiogram pattern with alternating QRS morphology and axis.^3,4^ Bogun et al. reported that a shared isthmus with different exit points may account for up to 40% of bimorphic VTs in ischemic cardiomyopathy.^5^ Therefore, the multiple exit sites from re-entrant circuits would be associated with these changing QRS morphologies^6^; however, three more options should also be considered: whether more than one arrhythmia is occurring, whether fusion with sinus beats is occurring, or whether tachycardia originates within or in the proximity of the His–Purkinje conduction system.^6–9^ In the current tracing, it is considered noteworthy that, every time the MDP appears, there is a consistent and immediate change in the QRS complex morphology. This observed pattern could theoretically represent either (1) a functional block within one pathway, resulting in activation through an alternative exit, or (2) a specific activation sequence within the scar substrate. Identification of the mechanisms for these changes is important when considering the feasibility of ablation as therapy.^1,10^

**Figure 2:**
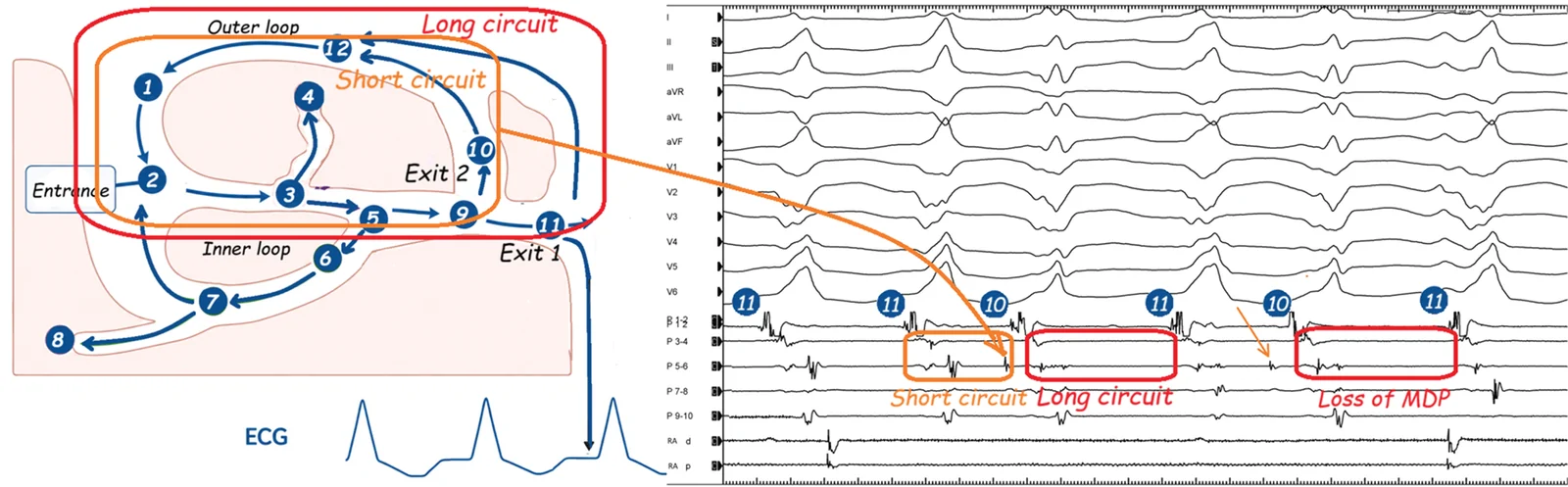
Intracardiac electrograms during ventricular tachycardia. *Abbreviations:* ECG, electrocardiogram; MDP, mid-diastolic potential.

The observed differences in timing and cycle length may arise from different exit sites of the isthmus (location “2” in **Figure 1**) rather than from a common isthmus (location “7” in **Figure 1**). The observed differences in timing and cycle length are more likely attributable to distinct exit sites of the isthmus (location “2” in **Figure 1**) rather than a single common isthmus (location “7” in **Figure 1**). Had the catheter been located within the common isthmus, the EGMs would be expected to be continuous rather than intermittent. The bimodal changes in QRS morphology support the presence of dual exits, while the shorter cycle length and distinct QRS morphology are more compatible with recordings from the catheter positioned in region 2. Therefore, these EGMs are most likely related to location “2” in **Figure 1**, which represents the only non-critical isthmus of the short VT circuit **(Figure 2)**, and are not associated with tachycardia termination. Even with ablation, they would be expected to reflect cycle length prolongation and a transition from bimorphic to monomorphic VT rather than true termination. Simply, the EGM morphology and location within the VT cycle allow electrophysiologists to understand the scar location and relation to the VT circuit.^11,12^
