# Erratum: Separate Electrograms and Morphologically Distinct QRS Complexes During Ventricular Tachycardia: Critical Versus Noncritical Isthmus

**DOI:** 10.19102/icrm.2026.17103

**Published:** 2026-10-15

**Authors:** Ozcan Ozeke, Dursun Aras, Serkan Topaloglu

**Affiliations:** 1 Department of Cardiology, University of Health Sciences, Ankara Bilkent City Hospital, Ankara, Turkey; 2 Department of Cardiology, İstanbul Medipol University, Istanbul, Turkey

The following corrections have been made to the article “Ozeke O, Aras D, Topaloglu S. Separate electrograms and morphologically distinct QRS complexes during ventricular tachycardia: critical versus noncriticial isthmus. *J Innov Cardiac Rhythm Manage*. 2026;17(8):6819–6821. doi: 10.19102/icrm.2026.17081.”

During manuscript production, the authors replaced several figures in the manuscript with new versions in response to an editor query but neglected to update several sentences associated with the old figures in the manuscript. The specific corrections are outlined below.

## Error 1

### Original sentence

“The observed differences in timing and cycle length may arise from different exit sites of the isthmus (location “2” in **Figure 1**) rather than from a common isthmus (location “7” in **Figure 1**).”

### Corrected sentence

“The observed differences in timing and cycle length may arise from different exit sites of the isthmus (location “10” in **Figure 1**) rather than from a common isthmus (location “3” in **Figure 1**).”

#### Correction

The sentence is now correct as presented in the online version.

## Error 2

### Original sentence

“The observed differences in timing and cycle length are more likely attributable to distinct exit sites of the isthmus (location “2” in **Figure 1**) rather than a single common isthmus (location “7” in **Figure 1**).”

### Corrected sentence

“The observed differences in timing and cycle length are more likely attributable to distinct exit sites of the isthmus (location “10” in **Figure 1**) rather than a single common isthmus (location “3” in **Figure 1**).”

#### Correction

The sentence is now correct as presented in the online version.

## Error 3

### Original sentences

“The bimodal changes in QRS morphology support the presence of dual exits, while the shorter cycle length and distinct QRS morphology are more compatible with recordings from the catheter positioned in region 2. Therefore, these EGMs are most likely related to location “2” in **Figure 1**, which represents the only non-critical isthmus of the short VT circuit **(Figure 2)**, and are not associated with tachycardia termination.”

### Corrected sentences

“The bimodal changes in QRS morphology support the presence of dual exits, while the shorter cycle length and distinct QRS morphology are more compatible with recordings from the catheter positioned in region 10. Therefore, these EGMs are most likely related to location “10” in **Figure 1**, which represents the only non-critical isthmus of the short VT circuit **(Figure 2)**, and are not associated with tachycardia termination.”

#### Correction

The sentences are now correct as presented in the online version.

The corresponding author expresses that they sincerely apologize for these errors and take full ownership and responsibility.

All corrections have been made to the online version of the article.

